# Book Reviews

**Published:** 1831-11

**Authors:** 


### CRITICAL ANALYSES.

Quæ laudanda forent, et quæ culpanda, vicissim

Illa, prius, cretâ ; mox hæc, carbone, *notamus*.—PERSIUS.

*Principles of Lithotrity: or, a Treatise on the Art of Extracting the Stone without Incision.* By Baron HEURTELOUP, Doctor of the Faculty of Medicine, Paris. *Illustrated with Plates of the Instruments used in Lithotrity.* 8vo. pp. 483. Whittaker, Treacher, and Co. London, 1831.

IT has rarely if ever happened that any new and very important improvement in practice has been suggested, without various disputes having arisen respecting the merit of the discovery. Lithotrity forms no exception to the general rule: many angry discussions have been entered into upon various points connected with this interesting subject. Claims and counter-claims to the invention of particular lithotritic instruments, and to superior dexterity in their practical management, have been confidently made on the one side, and as resolutely refused on the other. We shall not touch upon these contentions: they may, and indeed must, be interesting to the parties who have engaged in them; but the profession in general will be satisfied to know what has been done, and what may probably be yet further effected for the alleviation or cure of a formidable disease by means of lithotrity, and would not thank us for encumbering a subject of such vast importance in the science of surgery by any formal or detailed attempt to award, to each person, the precise amount of merit which is his due.

The author of the volume before us is well known to the profession for the great mechanical ingenuity which he has shewn in the improvement of some, and in the original construction of others, lithotritic instruments; while those who have been fortunate enough to witness his application of them in practice, have fully admitted his capability of employing them with a tact and delicacy which can only arise from a perfect knowledge of their various powers, and a well-grounded acquaintance with the anatomical structure of the parts upon which they operate.

Although every chapter of Baron HEURTELOUP's work must be interesting to the surgeon who wishes to make himself acquainted with the "principles of lithotrity," there are many we shall be compelled to pass over with but a cursory notice, as they do not admit of abbreviation. The introductory observations are important, inasmuch as they point out the advantages and precise objects of the practice of lithotrity. The first and most happy result of lithotrity will be to banish from the minds of patients the dread which the operation of lithotomy must always produce; and to induce persons labouring under stone in the bladder to have the calculi removed at the commencement of their formation, and not to wait till the bladder becomes diseased, and the chances of success consequently diminished.

"The second consequence of this operation, which results from the first, will be to prevent these patients passing many years of their lives in suffering and misery; for, not recoiling from an operation, the performance of which they know to be almost entirely free from danger, and generally little, or not at all painful, they will readily offer to submit themselves to it, without being compelled.

"If, however, lithotrity includes, in its general results, so many advantages over lithotomy, it also presents secondary ones, which are most important. A few observations on these two operations, compared with each other, will demonstrate this most unanswerably.

"Lithotomy requires to be performed in a favorable season; lithotrity may be performed at all times, with equal chance of success: the former requires that the patient should remain in bed for a month or six weeks after the operation; the latter needs no confinement, and often allows the patient to pursue his usual avocations. Lithotomy requires the patient to be kept on a rigid diet, while he who undergoes lithotrity is only moderately restricted; the recovery is often in the former case very tedious, whilst in the latter it is effected at once. Lithotomy often produces serious accidents, such as impotence, incontinence of urine, and urinary fistulæ; lithotrity, from its nature, cannot cause any of these consequences: the former, whatever may be the means employed, always requires a large and deep incision; the latter requires none. Lastly, lithotomy may cause almost instant death from hemorrhage; lithotrity cannot, under any circumstances, produce such a termination. We might carry this parallel still further, but the remarks we have just made, will, we think, be sufficiently convincing." (P. 3.)

Besides these advantages, the Baron mentions another of equal importance: from the nature of the operation of lithotrity, it allows trials to be made, "which, when performed with *care and prudence*," relieve the patient rather than increase his sufferings. Lithotomy admits of no half measures; once begun, it must be finished: "we can make a trial of lithotrity, we can never do the same with lithotomy." Two principal objections have been made to lithotrity, but the author contends they are both more speculative than real. It has been said that the instrument might break in the bladder, or that fragments of the calculus might remain, and become the nuclei of new formations. It is declared, however, that, in the many cures which have been effected by the lithotritic process, "no instrument has ever yet been broken;" and, admitting that this accident should happen, which it is conceived is scarcely possible, Baron H. thus replies to this objection.

"Lithotrity was invented to supersede lithotomy as often as possible: now supposing that in this operation the instrument should sometimes break, say once in twenty times, and that we cannot extract the broken portion through the urethra, we must perform lithotomy this once; still we shall, by lithotrity, have avoided lithotomy nineteen times out of twenty. Thus it is evident that this first objection is not a very serious one." (P. 5.)

As the patients who have been cured have had no return of their symptoms, although a long time has elapsed since the operation, and as in those who have been treated and cured by lithotrity, and who have subsequently died from some other cause, no vestige of any calculus could be discovered on examination, it appears that little or no fear need be entertained of fragments remaining in the bladder, from the inefficiency of the operation.

"But in admitting, that which is certainly possible, that one or two fragments do remain in the bladder of a patient who has undergone this operation, these fragments will give rise to fresh symptoms of stone, which will only require another and last application of the instrument, and which cannot produce any great inconvenience.

"If, besides, we bear in mind that lithotrity must not be considered in an absolute manner, but relatively to lithotomy, we shall say that fragments of stone, and even whole calculi, have been left in the bladder after this latter operation, which necessarily leads, not to the harmless re-introduction of the instrument of lithotrity, but to a second operation of lithotomy, an operation of much greater severity.

"Thus lithotrity still maintains its advantages in every respect." (P. 6.)

Before the destruction of stones in the bladder, by an instrument passed through the urethra, could be undertaken with success, it was necessary to prove that a perfectly straight sound, of three or four lines in diameter, could be passed into that organ. It is now well known that a careful and skilful surgeon can introduce such an instrument into the bladder. We have seen the straight sound passed several times, with but little pain to the patient, by Mr. Mayo, and other surgeons; but we must observe, at the same time, that this step of the lithotritic process would certainly be attended with hazard to the patient, as well as perplexity to the practitioner, unless the latter possess much more than a common share of dexterity and anatomical knowledge.

Baron Heurteloup enters into a rapid sketch, for the purpose of giving some idea of the state in which lithotrity was in the hands of those who preceded him, and what it is now, after the attempts he has made to bring it to its present state. It appears from the Baron's statement, that, although many facts had been known in relation to a rude lithotritic operation, they led to no conclusions of much service to science until the year 1813, "when a Bavarian physician, Doctor Gruithuisen, constructed from them the basis of a mode of operating, the result of which has been the scientific cure of calculous patients by mechanical means, and without making any incision. Opposing facts to prejudice, he publicly, *before experienced persons*, performed catheterism with a *large straight sound* upon the living subject. Gruithuisen was rewarded for his ingenuity by a gold medal, of the value of one thousand francs, from the Institute of France. From this moment the skill of professional men, which had as yet effected little towards treating calculous patients without incision, received an impulse, and the annals of science soon announced the fruits of new conceptions. We must pass over the successive improvements that were suggested by different individuals, and proceed at once to the claims made by the author: it is due to him to observe, that the Institut de France acknowledged the important results of his inventions by several prizes, and particularly the great prize of surgery for 1828.

After shewing the imperfections of the lithotritic apparatus which was employed before he devoted his attention to the subject, the Baron gives a concise sketch of the four principal objects he had in view, in order to render it more safe and effective.

"Thus, in the first place, I have substituted for the 'perce-pierre, which only destroys calculi of a certain size, by making repeated perforations in them, and which requires frequent attempts and much searching in the bladder, a system of excavating, which only requires the stone, especially when nearly spherical, to be once seized, in order to break it into pieces.

"Secondly, I have advantageously substituted for the same instrument, another peculiarly well adapted for destroying flat or concave fragments resulting from the action of the excavating instruments, and flat stones, which from their shape are extremely unfavorable for the 'perce-pierre.'

"Thirdly, I have advantageously substituted for the inconvenient position of the patient on a common bed, a position which may be altered and adapted at will to the comfort of the patient, and the convenience of the surgeon, and, *cæteris paribus*, renders the operation more expeditious and more gentle.

"Fourthly and lastly, I have advantageously substituted for the unsteady support of an assistant to maintain the instrument during the operation, a firm and immoveable 'point d'appui,' (fixed point,) useful when operating with the 'perce-pierre,' but indispensably necessary when operating with my excavating instruments." (P. 38.)

These improvements, added to some new ideas with regard to the accessory treatment, such, for instance, as ascertaining the form of the bladder and stone before commencing the operation; that of injecting; that of producing the expulsion of fragments from inert bladders, or the extraction of them when lodged in the urethra; the means of discovering small stones or fragments in the bladder, by a different method from that generally pursued, complete all the lithotritic apparatus which has been invented by the author. His mode of operating, then, does not consist in the employment of one single instrument, which, however perfect, can never be equally applicable to stones of all shapes and sizes, but in the application *of a particular and competent system of means, the different parts of which are applicable to different cases.*

The next section of the work requires the particular attention of the young lithotritist: it contains an account of the examination of the urethra and the bladder, as regards their connexion with lithotrity.

An instructive sketch is given of the first symptoms of calculus, and of the subsequent symptoms and the physical properties of calculi.

Baron Heurteloup then enters into a detailed description of the various lithotritic instruments, and this subject occupies a considerable portion of the work. An abridged account of these instruments would be useless, and, in order fully to comprehend their mechanism and action, the plates must be consulted.

There are various circumstances which may contribute to the success of the operation of lithotrity, and some which render it difficult or altogether inadmissible. This part of the subject, of course, demands the especial attention of the lithotritist: he is obliged to bear in mind numerous circumstances, which are of little importance to the lithotomist; for, acting by dexterity, and not by force, he is to turn aside obstacles rather than to overcome them.

"*The consideration of lithotrity, with regard to the age*

*of the patient.*" Lithotrity is not so applicable to children as adults: in the first place, because the operation is more frequently successful when the patient comes under the care of the surgeon soon after the commencement of the disease, and it seldom happens that the symptoms of stone are detected early in children; and if we consider

"How small in young subjects is the passage through which we are to introduce instruments into the bladder, and through which also the fragments of the stone are to be expelled, if we also consider how difficult it is to keep a child sufficiently quiet and motionless during the operation, for the surgeon to employ all the delicacy of his tact, we shall readily conceive how difficult it must be to practise lithotrity in such young subjects, and how much those favorable circumstances which accompany the operation in the adult are diminished in the child." (P. 308.)

It must not be forgotten that a stone which might be considered small in the adult is large in the child; for, in lithotrity, the stone must not be considered as to its absolute size, but relatively to the power of the instrument which is to destroy it, and to the size of the canal through which the fragments are to pass. Baron Heurteloup observes, however, that the question is not exactly whether lithotrity is *practicable* in children, but rather whether, if performed, it offers *greater chances of success* than lithotomy; and this he doubts, when the stone has acquired the size of a filbert before the age of three years, or of a small walnut at the age of five, six, or seven. Facts, it is said, support the opinion that each year which brings the child nearer to manhood adds to the chances of success held out by lithotrity. In old people the operation is unattended with difficulty, provided the texture of the parts engaged in it continue healthy.

In the performance of the lithotritic operation, there are some circumstances relating to the constitution, or general state of the patient, that merit attention. A great degree of obesity sometimes increases the difficulties of lithotomy, but has no influence upon lithotrity. Severe organic disease appears to the author sufficient reason to determine the impropriety of the operation, especially if the stone be so large as to require it to be attacked several times.

"Lithotrity, in common cases, seldom produces fever or organic derangement; but an unhealthy subject, particularly one in whom some of the vital organs are much diseased, is more liable to some sympathetic affection, resulting from the more or less irritation caused in the urinary system by the manœuvres which lithotrity renders necessary. If, however, the stone is small, and can be broken in two or three operations, we are of opinion that the patient may be subjected to the operation with every chance of success; but we are also of opinion that it ought not to be performed unless the stone causes great pain to the patient. It remains, however, with the surgeon to weigh well the advantages and disadvantages of lithotrity in these cases before rejecting or deciding upon its performance; but we would particularly impress this consideration, that the operation, when performed in these cases with the greatest care and precaution, may produce some sympathetic excitement, which generally settles upon the affected organ.

"Whatever may be the accidents supervening to lithotrity in such cases, these are not to be compared to those following lithotomy, which operation ought never to be performed in such patients. Thus we find in lithotrity a resource attended at least with many chances of success, and therefore applicable to cases which would inevitably terminate fatally, if submitted to the operation of lithotomy." (P. 316.)

Certain states of the urethra are unfavorable for the performance of lithotrity: the size of the canal may be too small, and although attempts may be made to dilate the urethra with bougies, cases so treated have not afforded satisfactory results. Strictures of the urethra may render lithotrity difficult or impracticable.

"It is impossible to decide until the canal is dilated as much as it admits of. We must, therefore, in these cases of stone combined with stricture, when there is any doubt about lithotrity, first treat the stricture; and we may do this the more readily, as at any rate this treatment will be beneficial to the patient, even should we afterwards find that the operation is admissible. If, on the contrary, from the diseased state of the bladder, and the size of the stone, we at once decide that lithotrity ought not to be attempted, it will then be better to perform lithotomy at once, provided the stricture be not so complete as to prevent the introduction of the staff." (P. 327.)

A large scrotal hernia embarrasses the operation, "inasmuch as it throws the canal to one side, and prevents our placing the instruments in the position we may judge necessary." Stones lodged in the urethra give rise to great, and sometimes even insurmountable, difficulties. If the mucous membrane of the urethra is soft and fungous, the operation will be less successful; for the rough fragments of the stone adhere to the membrane, which does not, in such a state, offer sufficient resistance. In some, although but few subjects, the urethra is so extremely sensitive, that the introduction of a gum-elastic sound produces great pain: sometimes this sensibility will continue in spite of every means, but it generally diminishes, or ceases altogether, by the daily introduction of flexible bougies, which should not be large enough to distend the canal, for such sensitive patients cannot bear too great extension. The state of the prostate gland has great influence on the degree of facility with which lithotrity may be performed. If it be enlarged, it impedes the operation, from its thickness and breadth. Sometimes the prostate forms a considerable cul de sac, in which the instrument may be engaged, and cannot be disengaged without depressing it considerably, or making it turn gently upon itself. Different malformations of the bladder are mentioned, which render the operation of lithotrity difficult, or even impossible. Simple mucous catarrh of the bladder does not prohibit the operation of lithotrity; but those patients are not fit subjects for it who have a muco-purulent discharge, and especially if it be attended with a hemorrhagic disposition, for this kind of catarrh is generally found in those who have suffered from stone for a long period, and in whom, consequently, the calculi have acquired a large size.

"As lithotrity can only restore calculous patients when the fragments of the stone may be expelled by the action of the bladder, the contractile power of this organ ought to be ascertained before deciding upon the operation. Thus a complete and permanent paralysis, arising from some injury to the medulla spinalis, and accompanied with symptoms which denote that the other organs participate in this paralysis, at once decides the impropriety of lithotrity, for the fragments will not be expelled.* The evil is not, however, very great, for in these cases, the stone does not generally cause much pain, and in the state of partial insensibility in which the patients are, they care little about being relieved from an inconvenience which is diminished by the sedentary life they are obliged to lead.

"Between, however, a state of perfect insensibility and complete loss of contractility, and the healthy and natural state of the bladder, there are many intermediate degrees, which may give rise to some special considerations.

"If the non-contractile state of the bladder be so great as to lead us to conclude that the fragments will be passed slowly and with difficulty, we must take into consideration the size of the stone; for if it is large, it is probable that the bladder will not be completely emptied of all the fragments by its natural expulsive power. If, on the contrary, it is small, the fragments may more easily be extracted, if they cannot be expelled." (P. 342.)

But if the want of contractility of the bladder be moderate, and if the patient void his urine freely, though he does not completely empty the bladder, lithotrity is recommended, even if the stone be large, "for the operation, in these cases, has the salutary effect of giving energy to the bladder, and

* "When this was written, the evacuating sound was not in my possession."

causing it to expel the fragments with sufficient power. A softened and fungous state of the mucous membrane of the bladder, causing some degree of hemorrhage on the slightest touch of the instrument, is unfavorable to, but does not absolutely prevent the operation.

"A varicose state of the veins, at the neck of the bladder, is also an unfavorable circumstance to the rapid progress of lithotrity. It does not prevent the introduction of the instrument, but it impedes the passage of the fragments.

"Lastly, a great degree of organic derangement, whether in the bladder itself or in the surrounding parts, may render lithotrity difficult, or forbid it altogether. We shall not stop to detail what all these changes may be, but we will point out the nature of some of them, in order that what we allude to may be understood. For example, a malformation of the pelvis, an exostosis from the interior of this cavity, unnatural adhesions of the bladder, hydatids, an enlarged ovary, or pregnancy, may alter the form of the bladder, and impede the operation." (P. 346.)

Difficulties may be occasioned by too great contractility of the bladder; and the Baron is of opinion that if the stone is very large, the operation is prohibited, unless this morbid state of the bladder can be remedied by appropriate means, as antiphlogistics or sedatives.

The author relates numerous cases in which he has performed lithotrity, and adds to most of these cases various instructive remarks as to particular points of the operation.

At the termination of the work, very well executed lithographic plates of the different instruments are given: the explanation of the plates is rendered additionally interesting by not being confined to a mere description of their mechanism. Their mode of action is also clearly illustrated.

Although only a very limited number of our readers may choose to undertake the operation of lithotrity, a general sketch of the leading principles which relate to it must be interesting to every practitioner. The subject of lithotrity is one which has excited almost as high a degree of public as of professional attention, and every medical practitioner is expected to be conversant with its general bearings, even if he may not profess to take upon himself the responsibility of operating. We confess we are far from wishing that the operation of lithotrity should be undertaken by surgeons in general: no man, at least, can be even moderately successful in his first attempts, who has not *frequently* seen it performed by some master of the lithotritic art: it is, indeed, not to be expected that sufficient information can be conveyed by written precepts to make a surgeon an expert lithotritist.

Book-learning can never fully teach us all the steps and niceties of a delicate and complicated operation. We make these remarks from a sincere wish that the character of lithotrity may not unjustly suffer, which it necessarily must if the operation should fall into the hands of those who have not carefully studied the mechanical powers of the various instruments employed, the different expedients that are required in different cases, and who have not duly prepared themselves by careful and repeated attention to the manœuvres of a well-practised lithotritist.

Baron Heurteloup's name is identified with the subject of lithotrity, from the many important improvements he has suggested in the apparatus employed, and the great dexterity he has shewn upon numerous occasions in the performance of the operation. The reputation he acquired in France has been confirmed by the suffrages of the first surgeons in this country, who have often given him the best proofs of their confidence by committing patients to his charge.

---

*Medico-Chirurgical Notes and Illustrations.* Part I. *On some Dangerous Affections of the Throat, which induce Sudden Death by Suffocation; on Strictures of the Œsophagus, and the Dangers of the Bougie; on the Cure of the Falling Down of the Bowel in Grown Persons; Anomalies in Rupture Operations, &c.* By R. FLETCHER, Esq., Surgeon to the General Infirmary at Gloucester, &c.—4to. pp. 146; with Plates. Longman and Co. London.

ALTHOUGH we have, in general, no reason to complain that the practical riches of men of experience are, like gold in the hands of a miser, of no advantage to the state, we could wish that such publications as this from Mr. FLETCHER were more frequent than they are. What kind of information can be more useful to the practitioner, or instructive to the student, than a plain relation of cases of diseases, in which success and failure are both stated with equal sincerity, and in which the mind of the reader is not drawn from practical facts to the more seductive, but infinitely less profitable, discussion of hypothetical speculations? Such is the kind of information bestowed upon the profession by Mr. Fletcher, and hence the great value of his work: it consists of a series of "Notes," each containing several examples of some important surgical disease, and most of the cases are followed by reflections from the author, in which he carefully scrutinizes the treatment that had been adopted, and not unfrequently criticises with much severity the practice followed even by himself.

The first "Note," (for such is the unassuming title of the different sections of the work,) is "*on the Sources of the Spasm of the Glottis*," a formidable affection, whenever and however it may arise, as the life of the patient is not unfrequently destroyed by it, when no danger is apprehended. It is believed by many competent judges that the process of suffocation from affections of the throat is often effected by the spasmodic action of the muscles of the glottis; and cases are recorded, and Mr. Fletcher adds to their number, in which there is, in his opinion, sufficient evidence to render this a fair and reasonable conclusion. The evidence adduced to support this opinion, which has been contested by a few pathologists, consists in the undeniable fact, that in many of these cases of sudden death from suffocation, or an obstructed windpipe, no obstruction of a mechanical kind, either within that tube or on its outside, can be discovered; whilst we know that the rima glottidis is surrounded by muscles that are capable of violent convulsive actions, upon the application of irritants, by which the tube may be closed and life destroyed.

Mr. Fletcher compares the spasm of the glottis with those which affect the urethra, œsophagus, and rectum, "and which are called spasmodic strictures, and, like these, that of the glottis is a sympathetic affection, derived from an original disease, more or less distant." Spasmodic stricture of the glottis varies in its degree and duration: the highest degree must prove quickly fatal, no air being able to pass through the rima glottidis; the urethra is sometimes in the same state; when there is a spasmodic stricture of this canal, scarcely a drop of urine will pass beyond it. At other times, the degree of spasm of the glottis is moderate, and will last a considerable time, causing slight convulsive catches of the breath, alternating and connected with more regular difficulty of breathing, and produced in some cases by pressure on the larynx and diseases within that canal.

" The spasm of the glottis is not accompanied by fever, unless this is caused by any of the inflammatory affections which may excite the spasm itself, as cynanche tonsillaris, laryngea, and trachealis, the croup of children, or phlegmonous abscesses on the outside of the larynx. From the rarity of the spasm of the glottis, but more especially from its having most frequently but a short-lived existence, medical men do not often have an opportunity of witnessing its wild, peculiar, and distressing character.

" The following was written down soon after leaving the bedside of the patient.

" When first seized with spasm of the glottis, the patient starts up suddenly, tossing his arms in wild affright; an expression of terror, as if attacked by some dreadful enemy within, sits upon his countenance; the eyebrows are raised over balls that are starting from their sockets; the shoulders rise and fall, as, with an open mouth and incredible exertions, air is drawn through the nearly obstructed tube, with a singular and alarming sound. Not a word is uttered; enough of the understanding is left in this moment of terror, to induce a belief in the unfortunate patient that one would be fatal: but, as the violence of the spasm subsides, he tells you in monosyllables, as well as he can, that he has been nearly choked.

"This communication is sometimes made with a voice that causes you to start; a deep, unnatural growl rises from the throat; or it is a fearful broken whisper, that still bespeaks terror, though now on the decline. As this terror continues to fade, the expression of the whole man assumes a different character. Apprehensive of a return, he seizes the bedclothes with his hands, which before were tossing in the air, that he may not be taken unprepared; and with one or the other he will occasionally point to the thyroid cartilage, as the seat of all his sufferings. A pale, haggard, and subdued countenance, on which are seen a few drops of cold perspiration, is before you; the mouth half open; the breathing yet hard. The eyeballs, indeed, have in a measure retired within their sockets, but the eyes themselves, as the understanding rallies, wear a mingled expression of keen, watchful intelligence, which follows your every movement, with restless anxiety." (P. 2.)

Fifteen cases are related of spasm of the glottis from different causes, of which five were fatal. That the patients who survived had the same affection as those who perished, is inferred from the fact that their symptoms were precisely alike. In the first case, the patient had complained for some weeks of a pricking pain and difficulty in swallowing; the breathing was afterwards occasionally impeded; she had a constant pain in her throat, neck, and side of the head; there was some swelling, with hardness and rigidity, of the muscles of the neck and lymphatic glands; pulse weak and slow. No solids could be swallowed; liquids passed with great difficulty, and in small quantities at a time. Large quantities of mucus in the throat caused great annoyance; the least movement of the head was intolerable. In about a month from her admission into the Gloucester Hospital, she died suddenly, her breathing having previously become alarmingly serious. The only morbid appearance found on dissection was a large ulcer in the œsophagus, about an inch below the situation of the cricoid cartilage; larynx free from disease.

"Case II. —— Takell, aged fourteen, was admitted into the Gloucester Infirmary, for a bronchocele which had existed for seven years.

"Of late, she said, her breathing had become considerably affected, especially at times. From the beginning of an examination of the tumor, (during which she complained of its being exquisitely tender to the touch,) her breathing became much affected, and towards its conclusion it assumed the character of the spasm of the glottis, with which I was not then much acquainted. She said the tumor had been frequently painful, but of late almost constantly so; that at such times it would not bear touching, and that it has recently increased in size.

"My note taken at the time says, 'she draws in air with uncommon difficulty, whilst handling the tumor, (which is painful to the touch, but not hot,) her countenance has a pallid and anxious expression; she has never menstruated, but her breath is perfectly good.

"'She is to be freely leeched daily till the pain and tenderness be subdued, and then a small seton is to be passed beneath the skin which is over the situation of the right lobe of the thyroid gland, but it is not to enter the substance of the tumor.'

"The bite of the leeches produced the same train of extraordinary symptoms which followed the manual examination: she sprung up in bed, throwing the leeches from her, at the same time raising the shoulders, and tossing her arms about wildly.

"In this position, with an open mouth, and protruded eyeballs, she drew in air with a laborious exertion, quite astonishing; and a frightened expression and haggardness of feature painful to look upon.

"The leeches were repeated three times, always followed by a repetition of this paroxysm. The nurse spoke of the great tenderness of the tumor, when handled in leeching; but there was no inflammatory redness of the skin, or increased temperature. The seton was passed. From this period there was certainly no amendment; the remainder of her life was a succession of the struggles to get air, and, as swallowing increased their violence, she attempted to take food no more, and died before the preparations for bronchotomy could be completed.

"On examination of the tumor, there was found a cyst, which arose from the centre of the right lateral lobe of the thyroid gland, which contained about a small teacupful of a fluid resembling ink. The lateral lobes themselves were considerably enlarged, but the right the most so.

"The larynx was perfectly healthy in its appearance, the membranous covering of the epiglottis, sacculi laryngis, and chordæ vocales, was totally free from inflammation, or its effects." (P. 5.)

Case III. is an example of spasm of the glottis from the irritation of a strumous or chronic abscess of the pharynx.

"—— Hopton, aged nine years, was brought in his father's arms for my advice, on account of the symptoms now to be described.

"The poor child, with his mouth half open, breathed slowly with a wheezing noise, and in a slightly laborious, though regular, manner.

He spoke with reluctance, and then in a grave whisper. His father said, he has complained of a pain in the throat, especially in swallowing, and also of pains about the sides and back of his head; but that he would not have brought the child under these circumstances alone, had he not been induced to do so from an attack of an extraordinary kind, which occurred to the boy in the night. He was suddenly seized with great additional difficulty of breathing; he threw out his arms in a convulsive manner, the eyes stared with a wild fulness, and he laboured to draw in air, in a way as to frighten himself and wife. It was a long, deep inspiration; but he held his breath so long afterwards that they thought he was dead; yet after awhile he would inspire again, but so reluctantly, that it seemed as though he did not like to part with the air, fearful that he should obtain no more.

"This mode of breathing happened only when he slept, which he always did in nearly an upright position." (P. 6.)

In this case no swelling or inflammation could be detected of the tonsils, uvula, or in the pharynx; there were slight appearances of mucus or lymph in the fauces. The boy had an emaciated appearance, and, from his general strumous character, ulceration of the larynx was suspected. Upon a subsequent examination, an abscess was detected low down in the pharynx: the abscess was opened, and a quantity of thin and imperfect pus discharged. For about a day afterwards, the breathing was almost natural. An attack of spasm again occurred, in consequence of the abscess filling with matter: it was opened a second time, and a teacupful of bad pus discharged: the breathing was instantaneously and completely relieved. The spasm did not again return, but the patient died some weeks afterwards, from strumous disease of the lungs.

The fourth case terminated fatally; spasm of the glottis being induced, as in the last instance, from the irritation of a chronic abscess of the pharynx.

"This man was a patient of one of the former surgeons of the General Hospital here. It appears little was done for him, because the affection in his throat was not known or suspected. It is nevertheless true that his life might have been saved, had the surgeon taken the necessary precaution of obtaining as much knowledge with his finger as it could furnish him: a speculum would have controlled the mouth, so as to have admitted a deliberate examination of the lower position of the pharynx, and a curved pharyngotomos would have penetrated the abscess." (P. 8.)

Case V. The patient had cynanche tonsillaris, to which he had been accustomed for years: "for two days he appeared to be doing as well as persons with sore throats usually do; but, on the fourth, a sudden and convulsive difficulty of breathing came on, as he was sitting up in bed, and he fell backwards, instantly suffocated, before assistance could be obtained." The body was not inspected.

CASE VI. "*Cynanche Tonsillaris, with Abscess, exciting Spasm of the Glottis.*" Mr. Fletcher was summoned in haste to see this patient, who was said to be in danger of suffocation.

"The fauces of Mr. S. were swollen to an unusual degree, the velum pendulum palati and the tonsils appeared to have advanced into the mouth, forming a complete barrier, and closing entirely the communication between the latter and the pharynx; whilst a slight undulating line could be scarcely traced, marking the boundaries of the tonsils, as they rested heavily on each other. He was breathing hard, but not spasmodically.

"There was a slight bulging of the velum pendulum palati just above the right tonsil, into which I plunged the pharyngotomos, and a torrent of pus was poured upon the floor. Shortening the lancet, the tonsils and velum were scarified freely, and after washing his mouth he spoke freely, and requested food.

"I saw Mr. S. no more: he recovered, though slowly, from a state of great exhaustion, spitting up daily, as reported, great quantities of mucus." (P. 10.)

Each of the cases which are detailed conveys a good practical lesson, and deserves to be carefully perused; but we are compelled to confine ourselves to the principal facts of the most interesting.

In the ninth case, violent spasm of the glottis arose from phlegmonous inflammation of the neck, but the patient was saved by the decisive practice adopted. Mr. F. was requested to drive with speed to a gentleman who was said to be suffocating: when he arrived, the violence of the attack had somewhat subsided, though the patient was still gasping for breath. The expression of countenance had the wild horror of one who had just escaped some frightful danger, and who was yet momentarily expecting its return. The spasm of the glottis was here plainly to be seen in the occasional convulsive catches of the breath.

"He grasped, as he sat, the bedclothes with both hands, and his mouth was open, drawing in air with great difficulty, his shoulders rising and falling as he did so, the eyebrows raised, the balls starting forwards, and a cold dew upon his pale face. As I approached the side of the bed, he let go the clothes with his right hand, and pointing to the front of the larynx, said in a hoarse and husky whisper, 'I shall be choked.'

"This gentleman had been subject to sore throats; indeed he had lately had one, the effect of cold on a night exposure.

"At present there was no vestige of any inflammation about the interior of the throat, though he now swallowed with difficulty; but on the exterior there was a general swelling across its middle, more especially on the left side, a little below the inferior cornu of the thyroid cartilage, deep and immediately over the carotid. The swelling had ascended high in the neck, making it look generally of a colossal size. At the point near the thyroid cartilage there was a redness, tenderness to the touch, and pain, marking the presence of phlegmonous inflammation." (P. 13.)

Fifty leeches were applied to the inflamed parts, and a strong purgative was given: the bleeding was profuse, and in three hours afterwards the respiration was infinitely more calm. In eight hours the breathing was nearly natural, the swelling of the throat greatly reduced, and the skin pale. The spasm of the glottis did not return, but in a day or two afterwards the patient brought up some offensive matter, and it was presumed that suppuration had taken place in the cellular membrane of the neck, and made its way into the pharynx.

The brevity and interest of the next case induce us to give it in Mr. F.'s words.

"*Spasmodic Stricture of the Glottis, from a small Irritable Bronchocele.* Elizabeth Cooke, of Corse, had a swelling on the left lobe of the thyroid gland, not bigger than a small walnut, which might be called bronchocele, or a tumor of this gland. She said it was painful when handled, but at ordinary times not so, except when she took cold, or was otherwise unwell, and then the tumor would increase in bulk, become regularly painful, and exquisitely tender to the touch. At such times too, she said, her breathing became strangely and unusually affected, so as to threaten suffocation, the fear of which brought her to my house for advice. Upon handling this swelling pretty freely, she put away my hand instantly, breathed convulsively, drawing air very distinctly and noisily through a narrowed glottis.

"The occasionally increased size of the tumor from cold, which raised the temperature and increased its sensibility, induced me to order a bleeding from the arm; but it appeared that attention to the stomach and bowels and the employment of tonics, especially the subcarbonate of iron, in doses of two drachms twice a day, was of the most service in diminishing the extraordinary irritability of this tumor." (P. 15.)

It appears from these cases that spasm of the glottis may be produced from the part sympathising with various distinct affections, more or less distant from the glottis.

The *treatment* of spasmodic stricture of the glottis consists in the speedy removal of its cause: if this be not done quickly and decisively, bronchotomy becomes necessary. When the causes of great difficulty of breathing in affections of the throat remain undiscovered, the surgeon is urgently warned not to depend upon a mere inspection of the parts by the eye: the fauces may appear sound, and yet in the depth of the pharynx may be concealed the true cause of the mischief.

"The most minute and careful survey should be made, with the hand and eye, of the interior and exterior parts of the throat, in all cases where the source of the spasm is doubtful.

"The pharynx in an adult is about two inches and a half long, as measured from the plane of the tongue to the commencement of the œsophagus; and nearly the whole of this portion of it may be explored with the finger, if the jaws are well fixed asunder by a proper speculum. The fore-finger of the right hand introduced between the molares, as far back as the corner of the mouth (on its right side) will admit of, may command the whole cavity.

"The examination should be done quickly and decisively, or otherwise the irritation of the finger will excite a renewal of these very spasms, or increase that difficulty of breathing, the cause of which it is meant to discover." (P. 20.)

In laryngitis or cynanche trachealis, the cause of the spasm is most hidden from view; but in either case it fortunately happens that the history and rapid course of the disease very clearly indicate its nature. The treatment of the foregoing case must, in the commencement, be vigorous in the extreme; for the rima glottidis or trachea will soon be choked with lymph, or the lining membrane be so thickened as mechanically to obstruct the air, which, of course, must greatly increase the danger from a supervening violent spasm. If acute inflammation of the fauces excite the spasmodic stricture of the glottis, whether advanced to suppuration or not, the most active local and general means should be immediately employed. If suppuration have taken place, a free opening in the abscess will remove the irritation and danger.

"Very powerful general means, and scarifying the inflamed part freely, will very frequently prevent suppuration; and surely such should be accomplished in all cases of extensive inflammation of the mucous lining of the fauces; for if we allow a large abscess of the tonsils to form, the passage for the air through the pharynx will be more or less obstructed mechanically, and vastly add to the danger of suffocation, should a spasm in addition take place.

"If however an abscess forms about the tonsil, notwithstanding the local treatment of scarification, or leeches to the outside of the throat, accompanied by a rising difficulty of breathing, no time should be lost; the patient's jaws should be well separated with a speculum, the tongue depressed, and an accurate examination be made of the fauces with the eye and finger.

"The abscess generally points, or rather slightly bulges through the velum, on the side inclining to that on which the tonsil is most affected.

"With the mouth fixed by a speculum, the fingers will usually be able to distinguish a fluctuation, and, although it may be somewhat obscure, yet the surgeon should be satisfied of the fact. Unless pains like these are taken, the pharyngotomus will be at work at random; several plunges may be made unsuccessfully with it, there being generally no such distinct pointing to lead the eye as in abscesses in a different structure.

"Surgeons have again and again punctured the tonsil, and retired from the patient with a belief that suppuration had not taken place, leaving him to an imminent risk of suffocation, the abscess remaining altogether untouched, and hourly rising in magnitude and danger. The examination had not been sufficiently minute to detect its real position. An abscess of the tonsil will sometimes take place on the back part, skirting it in a long and somewhat indistinct form, in the manner of the lip-like character of the gum abscess, as seen between the cheek and the jaw.

"The best instrument for opening the abscess of the tonsil is a broad lancet concealed in a sheath, called the pharyngotomus. With the aid of a speculum, which furnishes a good view, and a steady hand, it may be used effectively and safely; but there is mischief to be apprehended from carelessness, as well as from gross ignorance, even in this very simple operation: it would be the former, were a surgeon to point his instrument towards the angle of the jaw, and plunge it deep into the carotid, because he would not be at the trouble of gaining a good view; it would be something else, of an infinitely worse character, should he do this in ignorance of the situation of that vessel: and yet it has been struck more than once or twice, and the unfortunate patient sent to his account without warning by the very hand that should have saved him. Of course the pharyngotomus should be held in a straight line from one of the lateral incisors opposite the abscess, to the back of the pharynx, and striking the abscess only when the operator is certain that the mouth of the canula is resting fairly upon the spot he wishes to tap." (P. 22.)

When the tonsils become so large as seriously to interfere with the process of respiration and swallowing, and no local or general treatment is found capable of effecting their reduction to an innocent size, it becomes necessary to remove a large portion of them. Mr. F. does not think it requisite to remove the whole gland: from what he has seen, he believes this cannot be done without risking more bleeding than is desirable in so narrow and remote a spot.

For the removal of enlarged tonsils, Mr. Fletcher has been in the habit of using the ligature, with perfect success.

"This is done by first separating the jaws of the patient by means of a dilator, so that when the fauces are teazed by the fingers of the surgeon, the first shall not be bitten, nor the latter interrupted in his operation by the patient shutting the mouth.

"Before the dilator is fixed, a packer's noose, made of small whipcord, should be prepared. This is effected by making a single knot upon one end of the thread; this end, with the knot, is to be brought forward upon the other, so as to make a single noose upon itself, including the other, and to be drawn tight upon it close to the first knot. The free end of the thread is then to be passed through the ring of the simple instrument used by Mr. Chevalier, to tighten the knot. See his plate, 3d vol. Med. and Chir. Transactions. So far I go with Mr. Chevalier.

"The dilator is now fixed; the jaws are well open. The assistant seizes the tonsil with a double hook, on which hangs the noose. This last, with the ring appended to it, is held by the surgeon himself. Now the assistant must pull the tonsil from its loose bed in a diagonal direction across the mouth; its base becomes elongated, the noose is slipped over it, and carried by the fingers of the surgeon close around it; the ring is then run up to the gland, and the ligature tightened, which is repeated daily till the tonsil drops off, which it does in a few days.

"It may be supposed that this simple mode of noosing a tonsil could not be carried into effect where the base of the gland is very wide; but it is surprising how forcibly pulling out this part from its bed will contract the base of it, by elongation. Now and then it may so happen that the base would be too broad for the noose: I can only say, however, that I have never yet met with a tonsil with a base that would not yield to elongation, and, therefore, that could not be noosed in the way described.

"If the surgeon be bold and dexterous with his fingers, the whole operation is done with a rapidity and effect very different to the long and worrying mode pointed out by Cheselden, or the ingenious improvement of Mr. Chevalier." (P. 25.)

The subsequent "Notes" contain very interesting cases, and many excellent practical remarks on Strictures of the Œsophagus, and the Dangers of the Bougie; on the Falling Down of the Bowel in Grown Persons; on Chronic Abscess of the Cheek, from the Irritation of a Carious Fang of a Tooth; Anomalies of Strangulated Hernia; on Failures in Lithotomy; Dilatation or Aneurism of the Posterior Crural Artery; on a Chronic Encysted Tumor found near the Testes; on Effusion, Abscess, and Fungus from inflammation of the Testes; Deformity from a Burn, where the chin is bound to the Breast; and lastly, a Case of Imperforate Vagina is related. We have confined ourselves, in the present article, to a full account of the first paper in the volume: our readers will thus be better enabled to form an opinion of the work than if we had entered into a cursory view of all the subjects discussed in it: the subsequent "Notes" are, however, much too important to be passed over, and we shall from time to time give condensed notices of them in other departments of the Journal.

No surgical practitioner or student can peruse this first part of Mr. Fletcher's "Notes" without anxiously looking for the second, and confessing at the same time how deeply he is indebted to the author for the valuable practical hints he has already communicated. We most sincerely hope that Mr. Fletcher will still find relief, in continuing so laudable and useful an undertaking, from the "deep affliction" he slightly, but with much feeling, refers to in his Introduction. His professional abilities are proved by his work, and that nature has endowed him with a philosophic and well constituted mind is manifest from the courage he had to commence it, under circumstances that would have reduced many men to despondency and inactivity.

---

*A Systematic Arrangement of British Plants, by* WM. WITHERING, M.D. *Corrected and condensed; preceded by an Introduction to the Study of Botany, accompanied with Figures.* By W. MACGILLIVRAY, A.M., Member of the Wernerian Natural History Society, &c.—8vo. pp. 391; ten plates. Dove, London; Lizars, Edinburgh; Keene, Dublin.

So many are the alterations which the original work of WITHERING has undergone, in some cases for the better, in others for the worse, at the hands of his successive editors, that we think it scarcely just he should be allowed to enjoy the honour, or answer for the errors, which have thus been posthumously ingrafted on him. Withering, resting on the authority, and following the example of THUNBERG, abolished four of the Linnæan classes, and distributed the genera they contained among the first nineteen of the remainder. Lately, this attainder has been by his editors reversed, and here, as we think, very advantageously; the genera being again collected, the classes Gynandria, Monœcia, Diœcia, and Polygamia, resume their stations, with chastened honours. So much, likewise, has botanical diagnosis been advanced since the time of Withering, that the definitions of numerous genera, and the analytic scale to many sections, as planned by him, are no longer applicable; many of these improvements have been already introduced, but many more, it seems to us, remain to be adopted; and until Withering is rendered still less like himself than he has yet appeared, he can scarcely be esteemed in the van of science. However, it is perhaps more safe to follow than to lead, and Mr. MACGILLIVRAY has here, in this condensed edition, furnished young botanists with a portable and very useful manual, which we think, in some respects, superior to the too much abridged Compendium of SMITH, in which none but the essential generic characters are given; and, as a field-book, we esteem it preferable to the British Flora of HOOKER, by its retention of the essential generic characters, in a conspective form at the beginning of each class, as well as the more complete enumeration of the natural characters at the head of each separate genus: this we consider a very important feature, and one that is of great value to students: we would venture to recommend its adoption to the author of the British Flora, as well as to the editor of SMITH'S Compendium.

Still, if he dare not lead, we cannot but regret that following, as we think, too slowly in the wake of science, the editor should have retained many species in genera to which they now no longer ought to be attached; e. g. Actinocarpus is here registered as Alisma Damasonium, Centranthus as Valeriana rubra, &c. Valerianella is also called Fedia, and Conopodium Bunium, although it has long enough been shewn that our British plants have no right to the names they have borne, but belong to different genera: the true Fedia, indeed, belongs to a different class, not being, as our plant is, a triandrous, but a diandrous genus. The analysis of the class Pentandria, order Digynia, is also very faulty; for the old, and in some respects very convenient, and perhaps too hastily discarded, diagnosis by the involucra, which is here retained, wants entire remodelling, to omit the genera which have been lately formed on the seeds, and which are here in part introduced; for, to take but one instance, Phellandrium, which, when considered a genus distinct from Œnanthe, was rightly arranged in the section of the Umbelliferæ, "Involucris partialibus, universali nullo," while Œnanthe was, in a section, contradistinguished from the before named, by having the umbels "involucro universali partialique;" but it can never be easily discriminated by a student according to Mr. Macgillivray's plan, wherein Phellandrium, a plant having no general involucrum, is made a species of a genus; viz. Œnanthe, which is placed in a section said to be distinguished by having "*umbels* AND *umbellules* furnished with involucres," and is excluded from its old station, which section was known by having "the umbellules furnished with involucral bracteas, umbels naked." Nor will the objection be lessened by our being told that this is not the only genus thus circumstanced, nor the only species in this genus; as the more exceptions there are to the artificial index, especially when, as in this case, they are passed utterly unnoticed and wholly unprovided for, in every stage of the analysis, the more will our position be strengthened, that the scheme requires to be revised.

We recommend these, and also some other similar inconsistencies, to Mr. Macgillivray's particular attention, as they materially lessen the value of a very useful book: we likewise must beg him to allow his three orders, Filices, Hycopodineæ, and Equisetaceæ, to stand in the next edition merely as sections of the first order of the class Cryptogamia: it introduces needless complexity, to elevate them to the rank of orders, when they are so much more nearly allied to each other than to either of the remaining orders; and this the more especially as they consist, the one of two, and the other of a single genus only. But, if he should insist on their elevation, we are at a loss to know why the Marsileaceæ are omitted; indeed, we are not a little surprised that the two British genera, Isoetes and Pilularia, find no place in the present work.

